# Supplementary material for: High-mobility inertial domain walls driven by spin-transfer torque in a ferrimagnetic spinel oxide
Source: Nat Commun. 2026 Mar 31;17:4672. doi: 10.1038/s41467-026-71290-6 (PMC13201732; doi:10.1038/s41467-026-71290-6)
Supplement: Supplementary file 1 — Supplementary Information [file 41467_2026_71290_MOESM1_ESM.pdf]

Supplementary information

High-mobility inertial domain walls driven by spin-transfer torque in a ferrimagnetic spinel oxide

Mingxing Wu*, Shilei Ding, Laura van Schie, Shenghao Cai, Yuhao Qiu, Ao Du, Alexander E. Kossak, Rui Wu, Christian L. Degen, Xuegang Chen*, and Pietro Gambardella*

Corresponding Emails: mingxing.wu@mat.ethz.ch; xgchen@ahu.edu.cn; pietro.gambardella@mat.ethz.ch

This supplementary information includes:

Supplementary Note 1-11:

Note 1. DW structure and chirality in a larger region of the film

Note 2. In-plane magnetic-field dependence of DW velocities

Note 3. Discussion on DW motion direction

Note 4. Extraction of DW position from MOKE images

Note 5. Temperature increase due to Joule heating in the racetrack

Note 6. Theoretical limit of DW velocity in NCO

Note 7. DW dynamics formulation for ferrimagnetic NCO

Note 8. One-dimensional model for DW inertia effect

Note 9. Discussion on the influence of impedance mismatch

Note 10. DW velocity as a function of current density for various materials

Note 11. Energy consumption for DW racetrack devices

References 1-49

Supplementary Note 1: DW structure and chirality in a larger region of the film

To obtain sufficient data sets for the DW structure and chirality in NCO, we scanned a larger region of the film by NV magnetometry. We selected five locations for confined scanning with different DW orientations (DW1 to DW5). Figures S1a and S1b show examples of confined maps of B_{NV} and the extracted DW profiles for two orthogonal walls (DW3 and DW4). Although these two walls are orthogonal, they exhibit similar domain wall structures, indicating negligible magneto-crystalline anisotropy in the film plane. The parameters of δ, ψ extracted from B_{NV} fittings are summarized in Supplementary Table 1. The DW width is determined to be $\delta = 39 \pm 13$ nm. This is comparable to the typical width of a DW defined by $\delta = \pi\sqrt{\frac{A}{K_{\mathrm{U}}}}$, where A and K_{U} are exchange stiffness and uniaxial anisotropy constant, respectively, as shown in Supplementary Table 2. Meanwhile, the chirality $\psi = 1.7 \pm 0.4$ rad indicates a nearly pure Bloch wall configuration.

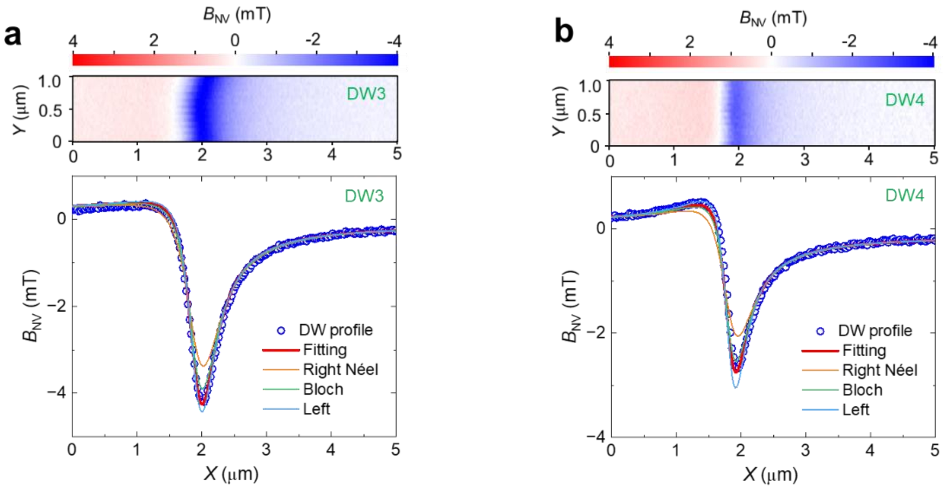

Fig. S1 The DW structure and chirality in a larger region of the film. **a**, **b** The confined scanning of B_{NV} and their corresponding fits for two orthogonal walls (DW3 and DW4).

DWs	δ (nm)	ψ (rad)
DW1	40 ± 20	1.5 ± 0.1
DW2	57 ± 6	1.5 ± 0.2
DW3	24 ± 5	2.3 ± 0.1
DW4	30 ± 9	1.9 ± 0.2
DW5	44 ± 11	1.4 ± 0.2

Table S1 Summary of the fitting parameters from the NV measurements.

Materials	Magnetism	DW width δ (nm)	DW type	Method	References
Ta/CoFeB(1)/MgO	FM	20	Bloch	NV center	[1]
Pt(3)/Co(0.6)/AlO_x(2)	FM	6	Néel	NV center	[1]
Fe	FM	64	/	Calculation	[2]
Co	FM	24	/	Calculation	[2]
$NiCo_2O_4$	FI	39 ± 13	Bloch	NV center	This work
Mn_4N	FI	25	/	Calculation	[3]
Pt/GdCo/TaO_x	FI	35	/	Calculation	[4]
Pt/CoTb/SiN_x	FI	31	/	Calculation	[5]
Bi-YIG	FI	44	/	Calculation	[6]
TmIG	FI	27 ± 6	Néel	NV center	[7]
Mn_3Sn	AFM	40	Néel	NV center	[8]
Cr_2O_3	AFM	42 - 65	Mixed Bloch-Néel	NV center	[9]
Mn_2Au	AFM	30	/	Calculation	[10]

Table S2 Summary of DW widths δ for various ferromagnets[1,2], ferrimagnets[3–7], and antiferromagents[8–10]. The DW width is extracted by $\delta = \pi\sqrt{\frac{A}{K_\mathrm{U}}}$, where A and K_U are exchange stiffness and uniaxial anisotropy constant, respectively.

Supplementary Note 2: In-plane magnetic-field dependence of DW velocities

To examine the possible DMI and SOT contribution for DW motion in NCO, we measured the DW velocities with Down | Up and Up | Down configurations as functions of the in-plane magnetic fields. The H_x and H_y denote the in-plane field that is applied along the racetrack and perpendicular to the racetrack. In the presence of DMI, DW tends to transit from a Bloch configuration to a Néel configuration due to the effective DMI field[4,11–16]. This internal field stabilizes a particular DW chirality. As a result, the DW velocity as a function of the external in-plane field exhibits a characteristic dome-like shape, where the maximum velocity occurs when the external field compensates for the DMI field[12]. The peak position therefore shifts away from zero, and the direction of the shift reflects the sign (chirality) of the DMI. The offset of the peak position provides a direct measure of the magnitude and sign of the DMI field. However, no discernible shift was observed in either H_x (Fig. S2a) or H_y (Fig. S2b) dependent DW velocities. This suggests that the DMI is negligible in the NCO film. Consequently, DW retains a predominantly Bloch character, consistent with the DW chirality measured using the scanning NV magnetometry.

On the other hand, in systems with spin-orbit torque (SOT), the DW velocity shifts in opposite directions under positive and negative currents when the H_y is applied[4,6,11–15]. This shift is proportional to the effective spin-Hall field. However, such a shift is not observed in the NCO DW racetrack, indicating the absence of the SOT contribution to the DW motion. Therefore, the DW motion in the NCO film can be attributed solely to STT. This agrees with the thickness-independent DW velocities in Fig. 3d in the main text.

Interestingly, the DW velocity remains constant under H_x whereas it reduces under H_y. This can be attributed to the influence of an in-plane field on DW width and, consequently, on the nonadiabaticity, given that nonadiabaticity inversely scales with DW width in terms of mistracking[17–19]. Other potential mechanisms, such as in-plane-field-induced Walker breakdown, only affects the DW velocity in the presence of H_x, but not H_y. Specifically, H_x lowers the Walker breakdown threshold, while H_y suppresses it. In this case, the DW velocity should change under H_x but not under H_y. This is in contradiction to the experimental observations, indicating that DW motion remains below the Walker limit under the applied in-plane magnetic fields. This is also consistent with our finding that $v_{avg} = \frac{\beta}{\alpha} u$.

The zero-field DW energy and width parameter in a perpendicularly magnetized film are determined by the competing exchange interaction and uniaxial anisotropy, and are given by:

$$\sigma_0 = 4\sqrt{AK_\mathrm{U}}\,, \qquad (S1)$$

$$\Delta_0 = \sqrt{\frac{A}{K_\mathrm{U}}}, \qquad (S2)$$

where A and K_U are the exchange stiffness and uniaxial anisotropy constant, respectively.

Applying an in-plane magnetic field H_P induces a Zeeman energy that modifies the wall structure. This effect can be incorporated into a modified anisotropy energy constant, expressed as[20]:

$$K' = K_\mathrm{U} + \frac{M_\mathrm{s}H_\mathrm{K}}{2}\cos^2\psi - \frac{\pi M_\mathrm{s}H_\mathrm{P}}{2}\cos(\psi - \psi_\mathrm{H}) = K_\mathrm{U} + \delta k, \qquad (S3)$$

where ψ_H is the angle of applied magnetic field relative to the x-direction, ψ is the angle of wall magnetization ($\psi = \pm\frac{\pi}{2}$ for a Bloch wall; $\psi = 0, \pi$ for a Néel wall), and $\delta k = \frac{M_\mathrm{s}H_\mathrm{K}}{2}\cos^2\psi - \frac{\pi M_\mathrm{s}H_\mathrm{P}}{2}\cos(\psi - \psi_\mathrm{H})$. NCO films exhibit Bloch wall chirality, and we therefore take $\psi = \frac{\pi}{2}$. The two additional terms in Eq. (S3) denote the demagnetizing energy due to magnetic charges on the walls with a demagnetizing field of $H_\mathrm{K} = \frac{\ln(2)}{\delta} t M_\mathrm{s}$ and Zeeman energy, respectively. The DW energy and width with the modified anisotropy become:

$$\sigma = 4\sqrt{A(K_\mathrm{U} + \delta k)} = \sigma_0\sqrt{1 + \frac{\delta k}{K_\mathrm{U}}}, \qquad (S4)$$

$$\Delta = \sqrt{\frac{A}{(K_\mathrm{U} + \delta k)}} = \Delta_0\sqrt{\frac{1}{1 + \frac{\delta k}{K_\mathrm{U}}}}. \qquad (S5)$$

For NCO, the uniaxial anisotropy field $\mu_0 H_\mathrm{U} = \frac{2K_\mathrm{U}}{M_\mathrm{s}}$ is estimated to be 2.8 T by taking K_U = 0.2 MJ/m^3 and M_s = 150 kA/m, which is significantly larger than the transverse demagnetizing field $\mu_0 H_\mathrm{K}$ (~ 80 mT) and the applied magnetic field $\mu_0 H_\mathrm{P}$. Therefore, we have $\delta k \ll K_\mathrm{U}$. The linear expansion of Eqs. (S4) and (S5) gives:

$$\sigma \approx \sigma_0 \left(1 + \frac{1}{2}\frac{\delta k}{K_\mathrm{U}}\right), \tag{S6}$$

$$\Delta \approx \Delta_0 \left(1 - \frac{1}{2}\frac{\delta k}{K_\mathrm{U}}\right). \tag{S7}$$

Inserting δk in Eqs. (S6) and (S7), we get:

$$\sigma = \sigma_0 + \Delta_0 M_\mathrm{s} H_\mathrm{K} \cos^2\psi - \pi \Delta_0 M_\mathrm{s} H_\mathrm{P} \cos(\psi - \psi_\mathrm{H}), \tag{S8}$$

$$\Delta = \Delta_0 \left[1 - \frac{H_\mathrm{K}}{2H_\mathrm{U}} \cos^2\psi + \frac{\pi}{2}\frac{H_\mathrm{P}}{H_\mathrm{U}} \cos(\psi - \psi_\mathrm{H})\right]. \tag{S9}$$

If a magnetic field H_x (Figs. S2a and 2c) is applied perpendicular to the wall magnetization, we have $\psi_\mathrm{H} = 0$. The wall magnetization angle ψ will change to minimize the DW energy. By calculating $\frac{\partial \sigma}{\partial \psi} = 0$, we get: $\sin\psi \left(\cos\psi - \frac{\pi H_x}{2H_K}\right) = 0$. If H_x is large enough to align the DW into Néel wall, $\sin\psi = 0$; otherwise, $\cos\psi = \frac{\pi H_\mathrm{x}}{2H_\mathrm{K}}$, which applies in our situation. Substituting $\cos\psi = \frac{\pi H_\mathrm{x}}{2H_\mathrm{K}}$ into Eq. (S9), we obtain:

$$\Delta = \Delta_0 \left[1 + \frac{\pi^2}{8 H_\mathrm{U} H_\mathrm{K}} H_\mathrm{x}^2\right]. \tag{S10}$$

If a magnetic field H_y (Figs. S2b and 2d) is applied parallel to the wall magnetization, we have $\psi_\mathrm{H} = \frac{\pi}{2}$. Similarly, minimizing the DW energy gives $\cos\psi \left(\sin\psi - \frac{\pi H_\mathrm{y}}{2H_\mathrm{K}}\right) = 0$. Here, the applied magnetic field is along the wall magnetization direction, which stabilizes the Bloch wall with $\psi = \frac{\pi}{2}$. Therefore, we have $\cos\psi = 0$. The DW width is then expressed as

$$\Delta = \Delta_0 \left[1 + \frac{\pi}{2H_\mathrm{U}} H_\mathrm{y}\right]. \tag{S11}$$

Figures S2c and 2d show the calculated Δ/Δ_0 as a function of the applied magnetic fields H_x and H_y. The behavior of Δ/Δ_0 differs under these two magnetic field directions. For H_x, the change in DW width depends on the anisotropy field H_K, because H_x tilts the wall magnetization and thereby modifies the demagnetizing energy. According to Eq. (S10) and Fig. S2c, the change Δ/Δ_0becomes weaker as H_K increases. In the limit $H_\mathrm{K} \gg H_\mathrm{x}$, the wall magnetization cannot be tilted, and DW width remains unchanged by H_x. In contrast, applying H_y does not tilt the wall

magnetization; instead, it aligns the wall magnetization to the field to lower the Zeeman energy. Consequently, the DW expands under H_y.

Quantitatively, the maximum change in DW width induced by a field H_x = 40 mT is estimated to be only 0.8%, and becomes even smaller for larger H_K. This small variation may explain why the DW velocity remains almost constant under H_x in Fig. S2a. However, a field H_y = 40 mT expands the DW width by up to 2.3%. The increase in DW width can reduce the nonadiabaticity, accounting for the observed decrease in DW velocity under H_y in Fig. S2b.

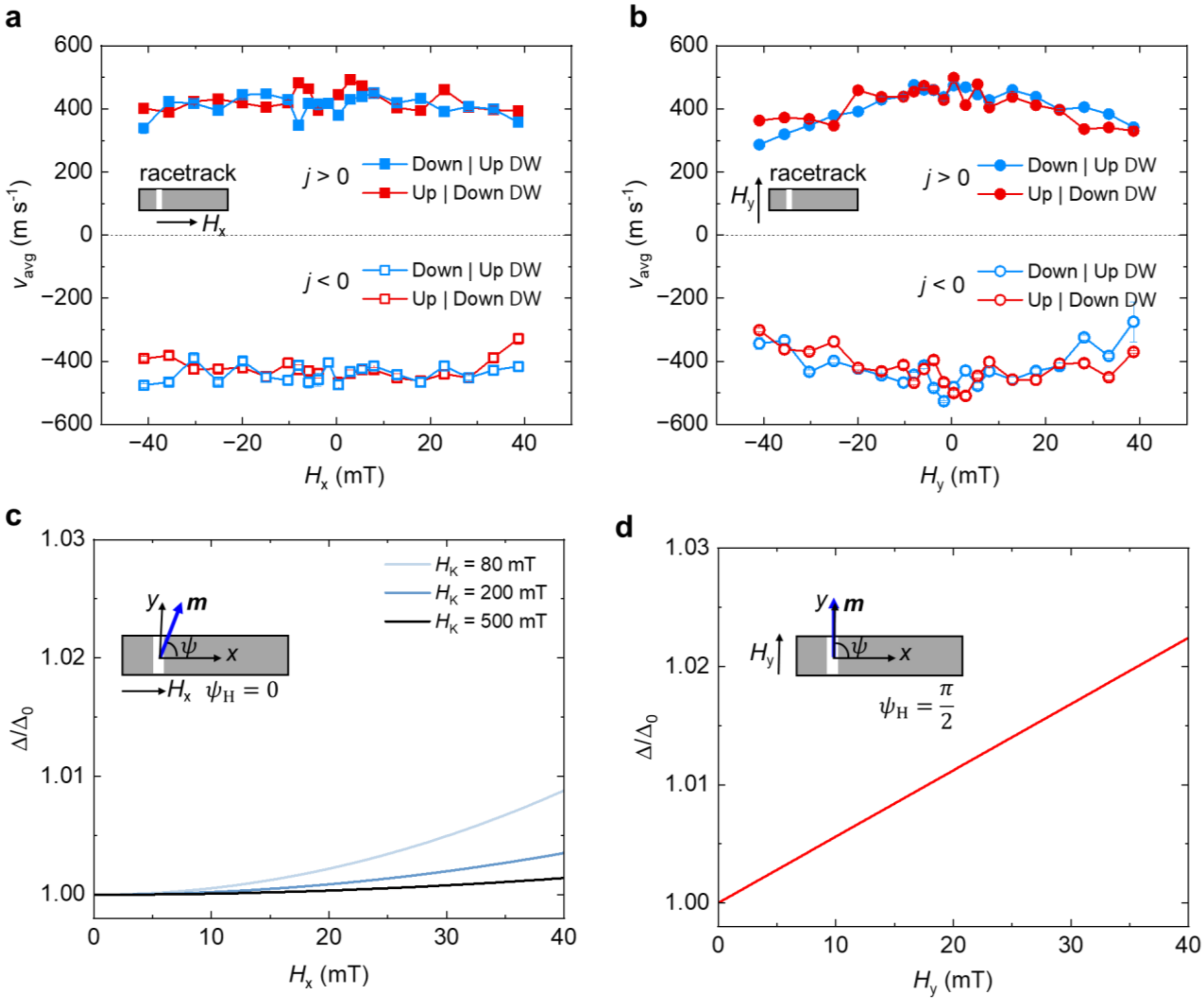

Fig. S2 DW velocities as functions of in-plane magnetic fields. a, b, H_x and H_y dependence of DW velocities for Down | Up and Up | Down domain configurations. The current density and pulse duration are set to 8.1×10^{10} A m^{-2} and 2 ns, respectively. The error bars are the standard deviations of the fits. c, d, Calculated DW width change Δ/Δ_0 as a function of H_x and H_y.

Supplementary Note 3: Discussion on DW motion direction

In the experiment, we find that DWs move in the same direction as the injected current, opposite to the direction of electron flow. This behavior has been observed in cases of STT-driven DW motion[12,21], which are attributed to a negative spin polarization of conduction electrons.

Here, we provide a schematic illustration of this phenomenon. As shown in Fig. S3, we consider an initial DW with Up | Down configuration (first panel). When a current is applied from right to left, conduction electrons flow oppositely (left to right) and become spin-polarized by the localized magnetization. If the magnetic film exhibits positive spin polarization (second panel case), the resulting spin-polarized current points upward. The transfer of angular momentum to the localized magnetic moments results in the DW motion towards the right—opposite to the current direction. Conversely, if the magnetic material has a negative spin polarization, the spin-polarized current drives the DW towards the left, resulting in DW motion in the same direction as the applied current (third panel).

In the case of NCO, the magnetic moments are carried by eight Ni ions (1.5 μ_B/Ni) on octahedral sites and eight Co ions (3.5 μ_B/Co) on tetrahedral sites, which are antiferromagnetically aligned[22]. The resultant ferrimagnetism is determined by the Co sublattice. However, the electronic spin states at the Fermi level are dominated by Ni, leading to a strong negative spin polarization. Theoretical calculations predict a negative spin polarization[23,24], which has experimentally been confirmed through magnetic tunnel junction measurements, yielding $P = -0.73$ [25]. Consequently, DWs in NCO are expected to move in the same direction as the injected current (third panel), in agreement with our experimental observations.

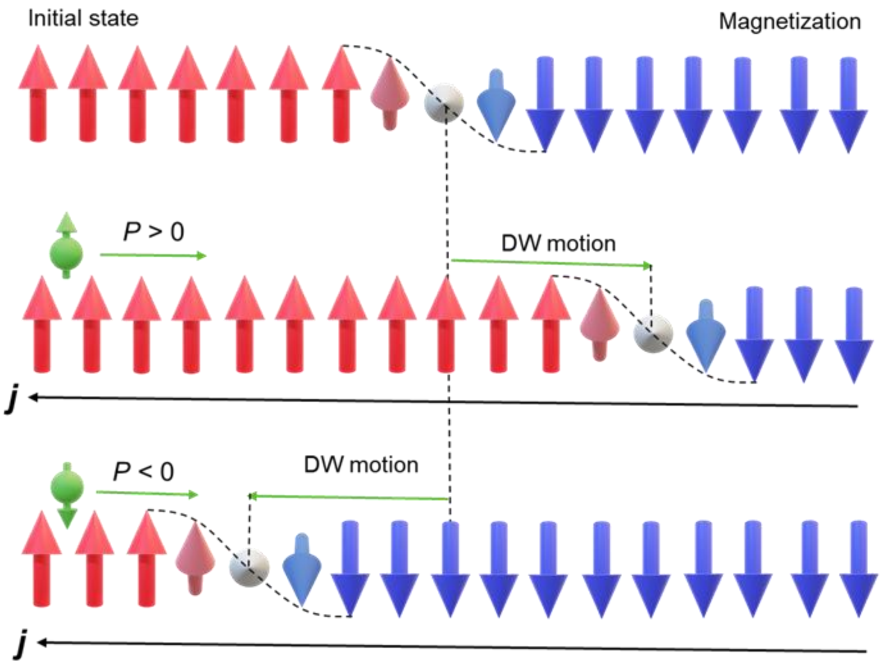

Fig. S3 Schematic illustration of the DW motion direction with respect to the injected current. The initial state (first panel) shows a DW with UP | Down configuration. Under a positive spin polarization (second panel), the DW moves opposite to the current direction, whereas it moves in the same direction as the injected current under a negative spin polarization (third panel).

Supplementary Note 4: Extraction of DW position from MOKE images

In the experiment, accurately extracting DW positions is crucial for determining the DW velocity. Here, we show the approach to extracting the DW position from the MOKE images. Figure S4a shows an exemplary sequence of DW motion under a series of pulses. We convert the image pixels into greyscale values and plot averaged horizontal line profiles, as shown in Fig. S4b. The wall center is identified at the half-height positions (indicated by dashed lines) of the rising and falling edges of the line profiles. The DW displacement in Fig. 3c of the main text is thus determined from the line profiles.

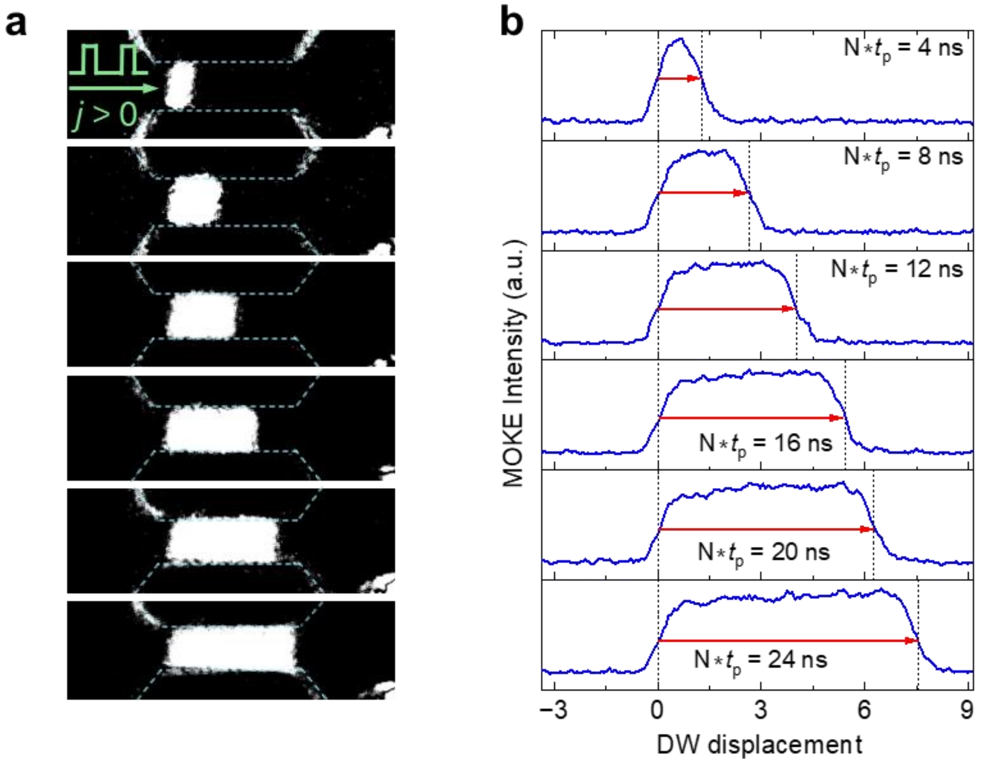

Fig. S4 Extraction of DW position from MOKE images. a, The MOKE images of DW motion under a series of pulses. b, The converted DW line profiles from MOKE images.

Supplementary Note 5: Temperature increase due to Joule heating in the racetrack

Here, we estimate the temperature increase due to Joule heating in NCO. The Joule heating Q_g during the pulse injection can be expressed by Joule's Law as $Q_g = I^2Rt$, where I, R, and t denote the amount of induced heating, applied current, sample resistance, and pulse duration time. Consequently, the temperature increase due to the Joule heating can be roughly estimated through the heating accumulation $Q_a = nC_{v.m}\Delta T$. where n, $C_{v,m}$, ΔT is the molar amount, molar heat capacity at constant volume, and temperature increase, respectively. In general, $Q_a \leq Q_g$ due to the heating dissipation during the pulse injection. One type of racetrack used in the experiments has dimensions of ~9 µm in length, ~3.4 µm in width, and 24 nm in thickness. The resistivity of NCO was measured to be ~ 810 $\mu\Omega \cdot cm$. Under the maximum applied current density of 2×10^{11} A m^{-2} with 2 ns pulse duration, the energy Q_g injected in the device is approximately 45 pJ. On the other hand, we use $C_{v.m}$ = 175 J mol^{-1} K^{-1} according to Dulong–Petit's Law. We ultimately obtain $\Delta T \leq 14$ K during the pulse injection. The magnetic transition temperature was reported to be 420 K for NCO[22]. Therefore, the temperature increase should have a negligibly small influence on DW motion.

Supplementary Note 6: Theoretical limit of DW velocity in NCO

In the case of an antiferromagnetically coupled sublattice, owing to the strong exchange field, the upper velocity limit of DW is governed by the magnon group velocity[26,27]. This can be derived from the spin-wave dispersion relation, and is given by $v_{\mathrm{g}}^{\max} = \frac{2A}{dS}$, where A is the exchange stiffness, d the lattice constant and $S = |S_1| + |S_2|$ the total angular momentum[6,26].

To estimate the theoretical velocity limit in NCO, we take the lattice constant d = 0.8 nm and A from the DW width: $\delta = \pi\sqrt{\frac{A}{K_{\mathrm{U}}}}$. Taking $\delta = 39 \pm 13$ nm, K_{U}= 0.2 MJ/m^3 [28], we obtain $A =$ $3.2 \pm 2.2 \times 10^{-11}$ J/m. The magnetic moments in NCO are carried by the antiferromagnetically coupled Ni (1.5 μ_B/Ni) and Co (3.5 μ_B/Co) sublattices[29]. Using g_{Co} = g_{Ni} = 2.2, we obtain S $= \frac{5\mu_B}{\gamma}/\mathrm{f.u.} = 3.62 \times 10^{-6}\ \mathrm{kg/(m \cdot s)}$. Based on these parameters, we estimate the theoretical limit of DW velocity as $v_{\mathrm{g}}^{\max} = 22 \pm 15$ km/s. The uncertainty here originates from the measurement of DW width using the NV magnetometry.

Achieving such a velocity limit requires a current density as high as 4×10^{12} A/m^2 according to the linear expansion of Fig. 3e. Although it remains challenging at present, future strategies, such as reducing magnetization (e.g., through Ni doping[28]) and increasing film conductivity, could further enhance the DW mobility, potentially enabling the observation of relativistic DW dynamics in NCO.

Supplementary Note 7: DW dynamics formulation for ferrimagnetic NCO

STT-driven DW dynamics can be generally described by the Landau–Lifshitz–Gilbert (LLG) equation, expressed as[30,31]:

$$\partial_t \boldsymbol{m} = -\mu_0 \gamma \boldsymbol{m} \times \boldsymbol{H}_{\text{eff}} + \alpha \boldsymbol{m} \times \partial_t \boldsymbol{m} - L_S(\boldsymbol{u} \cdot \boldsymbol{\nabla})\boldsymbol{m} + \boldsymbol{m} \times (\beta L_S \boldsymbol{u} \cdot \boldsymbol{\nabla})\boldsymbol{m}, \tag{S12}$$

where $\boldsymbol{m}$ is the unit vector along the magnetization, $\boldsymbol{H}_{\text{eff}}$ the effective magnetic field, $\boldsymbol{u}$ the spin-drift velocity, defined as $L_S \boldsymbol{u} = \frac{P\hbar}{2e}\boldsymbol{j}$, with P is the spin polarization, $\hbar$ the reduced Planck constant, $\boldsymbol{j}$ the injected current density. $\gamma = \frac{g\mu_B}{\hbar}$ is the gyromagnetic ratio where μ_B is the Bohr magneton and g is the Landé factor. $L_S = \frac{M_s}{\gamma}$ is the angular momentum density and M_s is the saturation magnetization. μ_0 is the vacuum permeability, α the Gilbert damping constant with $L_\alpha \equiv \alpha L_S$, and β the nonadiabatic coefficient. In ferromagnets, $L_S \neq 0$, and the magnitude of the spin-drift velocity reduces to $u = \frac{g\mu_B P}{2eM_s} j$.

Unlike ferromagnets, ferrimagnets possess antiferromagnetically coupled sublattices, which must be explicitly considered when deriving DW dynamics. To capture this behavior, two individual LLG equations— LLG ($\mathbf{m}_1$) and LLG ($-\mathbf{m}_2$)—can be formulated for the two sublattices, where $\mathbf{m}_1$ and $\mathbf{m}_2$ represent the respective sublattice magnetization directions. The sum of these two LLG equations yields an effective LLG ($\mathbf{m}$) equation, identical in form to Eq. (S12), but expressed using effective material parameters. Specifically, $M_s = M_1 - M_2$ and $L_S = \frac{M_1}{\gamma_1} - \frac{M_2}{\gamma_2}$, while α, β and P correspond to the effective Gilbert damping constant, the effective nonadiabatic coefficient and the effective spin polarization, respectively.

By applying these effective parameters, the one-dimensional (1D) model is applicable for ferrimagnets with antiferromagnetically coupled sublattice magnetic moments. Below the Walker breakdown, the analytical expression of STT-driven DW velocity for ferrimagnets is (Table 1 of Ref. [31]):

$$v = \frac{\beta}{L_\alpha} L_S u. \tag{S13}$$

Depending on whether a compensation temperature exists, ferrimagnets can be classified into two categories with different descriptions of DW dynamics. In the case of rare-earth transition-metal ferrimagnets such as GdFeCo[32,33], Gd_xCo_{1-x}[4,34], $Co_{1-x}Tb_x$[5], the magnetization compensation

temperature (T_M) occurs below room temperature due to the distinct temperature dependencies of the sublattice magnetizations. In addition, the Landé g-factors differ between rare-earth and transition-metal atoms, e.g., $g_{Gd} = 2$ and $g_{Co} = 2.2$ [5]. This leads to distinct gyromagnetic ratios and, consequently, an angular momentum compensation temperature T_A ($L_S = 0$) away from T_M. In this case, the DW velocity $v = \frac{\beta}{\alpha L_S}\frac{\hbar P}{2e}j$ diverges as the temperature approaches T_A. Only when the temperature is far away from T_A, $L_s \approx \frac{M_1 - M_2}{\gamma} = \frac{M_s}{\gamma}$ with $\gamma = \gamma_1 \approx \gamma_2$ and $M_s = M_1 - M_2$, the DW velocity becomes $v = \frac{\beta}{\alpha}\frac{g\mu_B P}{2eM_s}j$, similar to ferromagnets.

In NCO, the magnetic moments are carried by eight Ni ions (1.5 μ_B/Ni) on octahedral sites and eight Co ions (3.5 μ_B/Co) on tetrahedral sites, which are antiferromagnetically aligned. The sublattice magnetic moment of Co remains larger than that of Ni at all temperatures below the Curie temperature, and thus no magnetization compensation point is reached. This is supported by the magnetization–temperature (M-T) and coercivity–temperature (H_c-T) curves, as shown in Fig. S5. No dip in the M-T curve or peak in the H_c-T curve was observed, both of which are characteristic signatures of a magnetization compensation point. Moreover, Co and Ni possess nearly identical Landé g-factors ($\approx$ 2.2), meaning that the angular momentum does not compensate either. As a result, the L_S can be directly simplified as $L_S = \frac{M_1 - M_2}{\gamma} = \frac{M_s}{\gamma}$, which does not diverge in NCO ($L_S \neq 0$). The DW velocity is thus expressed as: $v = \frac{\beta}{\alpha}\frac{g\mu_B P}{2eM_s}j$, consistent with our description in the main text. This formulation is equivalent to that of ferromagnets, with the expression in terms of the effective material parameters, and thus remains valid across the entire temperature range.

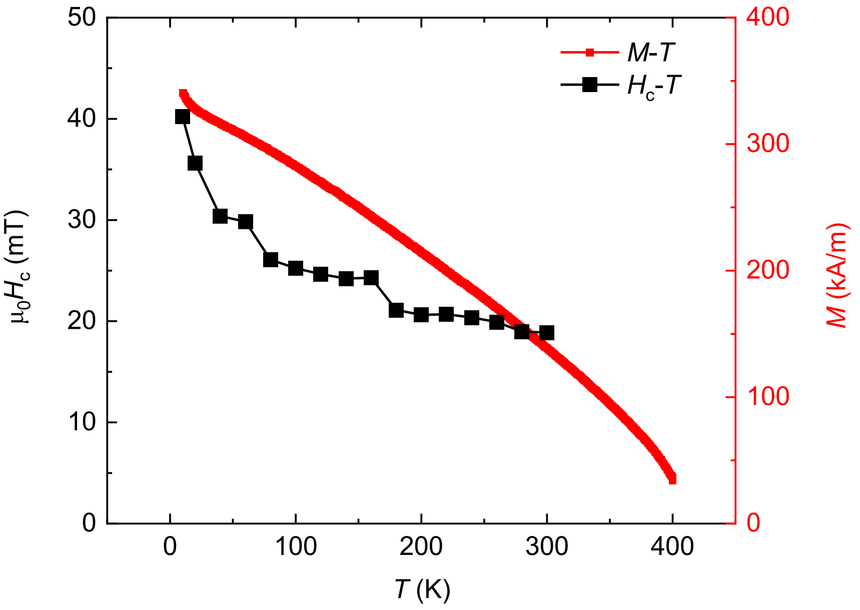

Fig. S5 *M*-*T* and H_c-*T* curves of 30 uc NCO. An out-of-plane magnetic field of 100 mT was applied for measuring the *M*-*T* curve. The coercive field H_c was extracted from *M*-*H* curves measured at different temperatures.

Supplementary Note 8: One-dimensional model for DW inertia effect

The DW inertia can be described using a one-dimensional model[30,35–38]. Here, the dynamic framework of a DW is depicted by two time-dependent parameters: the wall position $q(t)$ and magnetization angle $\psi(t)$, where ψ is defined as the angle between the magnetization vector in the wall center and $+x$ direction. $\psi = \frac{\pi}{2}$ denotes a Bloch wall, whereas $\psi = 0$ or π corresponds to a right or left Néel wall, respectively. The DW equations of motion are given as:

$$(1+\alpha^2)\dot{q} = -\frac{\gamma\Delta H_{\mathrm{K}}}{2}\sin(2\psi) + (1+\alpha\beta)u \tag{S14}$$

$$(1+\alpha^2)\dot{\psi} = \frac{\gamma\alpha H_{\mathrm{K}}}{2}\sin(2\psi) + (\beta-\alpha)\frac{u}{\Delta}. \tag{S15}$$

Here, α is the effective Gilbert damping constant, β denotes the effective nonadiabatic coefficient of STT, Δ is the DW width parameter (the physical DW width $\delta = \pi\Delta$), γ is the gyromagnetic ratio, H_{K} is the demagnetizing anisotropy field originating from magnetostatic energy. The positive spin-drift velocity u is defined along the electron flow direction. The DMI and SOT-induced effective fields are vanishing in NCO film and thus are discarded in the above equations. To obtain an analytical solution, the equations can be simplified by applying the first-order expansion of $\sin(2\psi)$ around $\psi = \frac{\pi}{2}$. In this way, the wall position q(t) can be described by the following differential equation:

$$(1+\alpha^2)\ddot{q} = -\gamma\alpha H_{\mathrm{K}}\dot{q} + \gamma\beta H_{\mathrm{K}}u \tag{S16}$$

For the initial condition, we assume a simple case: $q = 0$ and $\dot{q} = 0$ [38]. The DW acquires a velocity when a pulse is applied in the racetrack, giving

$$v_{\mathrm{on}}(t) = \dot{q} = \frac{\beta}{\alpha}\mu\left(1 - e^{-\frac{t}{\tau}}\right), \tag{S17}$$

where $\tau = \frac{1+\alpha^2}{\alpha\gamma H_{\mathrm{K}}}$ is a characteristic parameter for DW acceleration. If $\beta = 0$, the DW does not exhibit steady motion but instead undergoes precessional motion. When $\beta \neq 0$ and $t_{\mathrm{p}} \gg \tau$, the DW reaches a terminal velocity $\frac{\beta}{\alpha}\mu$ in steady state, which is proportional to the nonadiabatic component of STT. On the other hand, when $t_{\mathrm{p}} \sim \tau$, the DW does not have sufficient time to accelerate to the

terminal velocity. When the pulse is turned off, the DW does not stop immediately. Instead, an inertial effect emerges as the magnetization relaxes toward its equilibrium state. The DW velocity due to the inertial effect is given by

$$v_{\mathrm{off}}(t) = \frac{\beta}{\alpha}\mu\left(1 - e^{-\frac{t_{\mathrm{p}}}{\tau}}\right)e^{-\frac{t-t_{\mathrm{p}}}{\tau}}. \tag{S18}$$

Here, a factor of $\left(1 - e^{-\frac{t_{\mathrm{p}}}{\tau}}\right)$ is introduced to satisfy the boundary conditions and to avoid an abrupt jump. Consequently, the instantaneous DW velocity under a pulse with a duration of t_{p} is given by[38]

$$v(t) = \begin{cases} \frac{\beta}{\alpha}u\left(1 - e^{-\frac{t}{\tau}}\right) & \text{for } 0 \leq t < t_{\mathrm{p}} \\ \frac{\beta}{\alpha}u\left(1 - e^{-\frac{t_{\mathrm{p}}}{\tau}}\right)e^{-\frac{t-t_{\mathrm{p}}}{\tau}} & \text{for } t \geq t_{\mathrm{p}} \end{cases}. \tag{S19}$$

The DW acceleration can be obtained by taking the time derivative of velocity as $\frac{\beta}{\alpha\tau}u$, which scales with $\frac{\beta}{\alpha}$ and $\frac{1}{\tau}$. A large nonadiabaticity and short τ lead to faster DW dynamics. The average DW velocity can be determined from the total DW displacement after applying a pulse of duration t_{p} and is given by: $v_{\mathrm{avg}} = \frac{1}{t_{\mathrm{p}}}\int_0^\infty v(t)dt$. We have:

$$v_{\mathrm{avg}} = \frac{\beta}{\alpha}u\left(1 + \frac{\tau}{t_{\mathrm{p}}}e^{-\frac{t_{\mathrm{p}}}{\tau}}\right). \tag{S20}$$

Clearly, if $t_{\mathrm{p}} \gg \tau$, the traveling distance of DW during the acceleration and deceleration are equivalent. In this regime, the DW predominantly moves at the terminal velocity, and the inertial contribution becomes negligible. However, when t_{p} is comparable to τ, the inertial effect becomes more pronounced. This explains that the measured velocity varies with t_{p}.

Other works in the literature consider a finite initial DW velocity $\dot{q} = u$ induced by the adiabatic torque in response to an abrupt current pulse[35,37,39]. In this case, the instantaneous DW velocity in Eq. (S19) can be expressed as[37]:

$$v(t) = \begin{cases} \frac{\beta}{\alpha}u + (1 - \frac{\beta}{\alpha})ue^{-\frac{t}{\tau}} & \text{for } 0 \leq t < t_{\mathrm{p}} \\ (\frac{\beta}{\alpha} - 1)ue^{-\frac{t-t_{\mathrm{p}}}{\tau}} & \text{for } t \geq t_{\mathrm{p}} \end{cases}. \tag{S21}$$

The average DW velocity is calculated as:

$$v_{\mathrm{avg}} = \frac{\beta}{\alpha}u + \left(\frac{\beta}{\alpha} - 1\right)u\frac{\tau}{t_{\mathrm{p}}}e^{-\frac{t_{\mathrm{p}}}{\tau}}. \qquad (S22)$$

In our case, since $\frac{\beta}{\alpha} \gg 1$, Eq. (S22) is equivalent to Eq. (S20) under the approximation of $\frac{\beta}{\alpha} - 1 \approx \frac{\beta}{\alpha}$. The initial DW velocity has a negligible influence on the fitting. Therefore, Eq. (S20) is used to fit the inertia effect for simplicity.

Supplementary Note 9: Discussion on the influence of impedance mismatch

The two-terminal resistance of our device is about 1.5 – 2.6 kΩ for 30 and 10 uc NCO devices, which includes the racetrack and the contacting pad resistance. To investigate the influence of impedance mismatch on the pulse waveform, we compared the pulse shapes measured without and with a sample (Fig. S6a). Figures S6b and S6c show the results for 1-ns and 3-ns pulse shapes. These comparisons clearly indicate that impedance mismatch does not distort pulse shape during transmission through the sample.

Nevertheless, the magnitude of the pulse must be carefully calibrated because impedance mismatch causes pulse reflection. The reflection can be quantified by the coefficient:

$$\Gamma = \frac{R_{\text{load}} - Z_0}{R_{\text{load}} + Z_0}, \tag{S23}$$

where R_{load} is the sample resistance and $Z_0 = 50\ \Omega$ is the pulser impedance. According to this equation, if $R_{\text{load}} = 50\ \Omega$, $\Gamma = 0$, which means a perfect match without reflection. In this matched case, the voltage on the sample equals the set output voltage: $V_{\text{load}} = V_{\text{set}}$. When $R_{\text{load}} \neq 50\ \Omega$ ($\Gamma \neq 0$), V_{load} is not equal to V_{set} anymore and must be recalculated as,

$$V_{\text{load}} = V_{\text{set}}(1 + \Gamma) = V_{\text{set}} \frac{2R_{\text{load}}}{R_{\text{load}} + Z_0}. \tag{S24}$$

Figure S6d plots V_{load} as a function of R_{load}. As expected, $V_{\text{load}} = V_{\text{set}}$ when $R_{\text{load}} = 50\ \Omega$, and V_{load} approaches $2V_{\text{set}}$ if $R_{\text{load}} \gg 50\ \Omega$. In our experiment, we employed this method to determine V_{load} for each device to ensure the accurate calculation of current density.

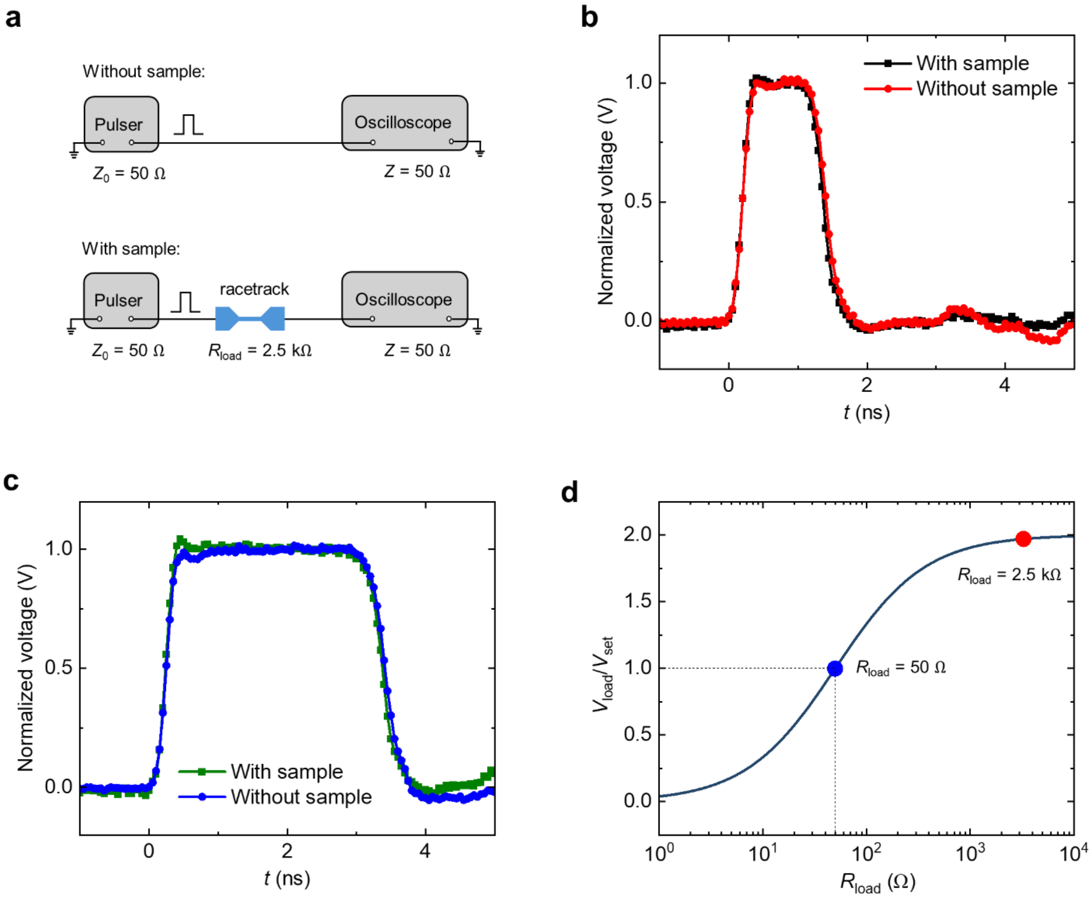

Fig. S6 Pulse waveform measurement. a, Schematic of the electrical circuit with and without the sample. **b, c,** Shape comparisons of 1-ns and 3-ns pulses with and without sample configurations. d, Plot of $\frac{V_{\text{load}}}{V_{\text{set}}}$ as a function of R_{load}.

Supplementary Note 10: DW velocity as a function of current density for various materials

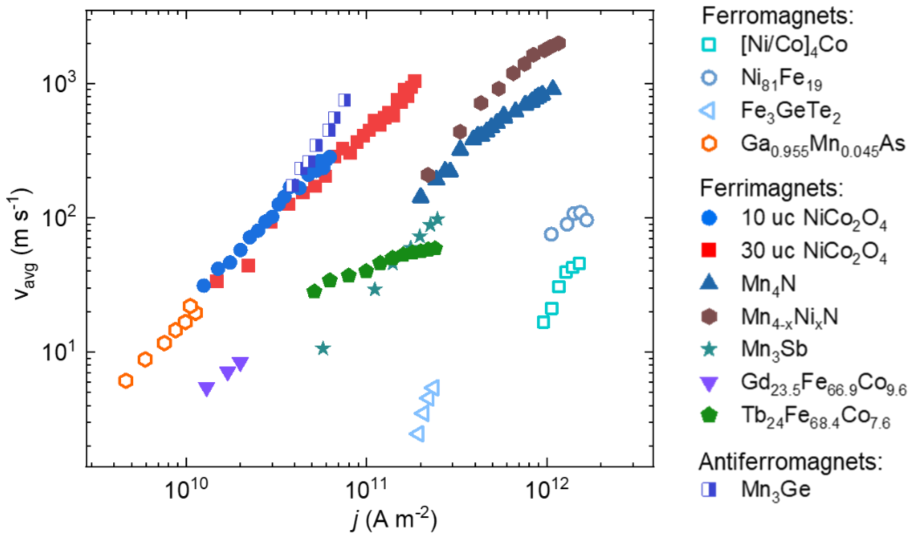

Fig. S7 DW velocity as a function of current density. The DW mobility in the main text is extracted from the linear fits.

Supplementary Note 11: Energy consumption for DW racetrack devices

DW mobility and operating current density are commonly employed to evaluate the performance and energy efficiency of DW devices[3,40,41]. However, these metrics do not account for the resistivity of devices, which significantly impacts the energy consumption in DW racetrack devices. To address this, Kumar et al[42] proposed evaluating the energy consumption using the energy required for 1 μm DW displacement, given by:

$$\xi = \frac{I^2 R t}{s}, \qquad (S25)$$

where I is the injected current, R is the device resistance, t is the pulse duration, and s is the DW displacement. This equation can be rewritten as:

$$\xi = \frac{j^2 \rho \mathcal{A} L}{v_{\text{avg}}}, \qquad (S26)$$

where ρ is the resistivity, $\mathcal{A}$ is the cross-sectional area, and L is the racetrack length. Clearly, ξ depends on the device geometry, which makes it challenging to compare the STT efficiency for driving DWs in different material systems (Fig. S8a). To solve this issue, we normalize ξ by the device volume. Accordingly, the volume-normalized ξ_V is defined as:

$$\xi_V = \frac{j^2 \rho}{v_{\text{avg}}}. \qquad (S27)$$

With this definition, ξ_V reflects the effective energy consumption per 1 μm DW displacement. It is proportional to the j, the mobility (j/v_{avg}), and ρ (Supplementary Table 3).

In Fig. S8b-d, we compare different normalization factors for evaluating DW device performance. Among them, only the volume-normalized energy consumption $\xi_V = \frac{j^2 \rho}{v_{\text{avg}}}$, shown in Fig. S8b, captures the intrinsic material properties independently of the device geometry. The energy consumption per device length, $\xi_L = \frac{j^2 \rho \mathcal{A}}{v_{\text{avg}}}$, shown in Fig. S8c, describes the energy required for displacing a DW associated to the cross-section of the material. The energy consumption per device cross section, $\xi_{\mathcal{A}} = \frac{j^2 \rho L}{v_{\text{avg}}}$, shown in Fig. S8d, describes the energy required for displacing a

DW across a device of length L, independently of its cross section. In all cases, we observe that NCO performs well relative to other materials. However, in Fig. S8b, 10-nm and 30-nm-thick NCO devices exhibit similar energy consumption, confirming that the influence of film thickness is effectively removed. Moreover, the enhanced DW performance of antiferromagnets relative to ferromagnets becomes unambiguous in Fig. S8b. Thus, the volume-normalized energy consumption serves as a robust guideline for identifying material properties and systems that enable high STT-driven DW mobility with minimal energy cost.

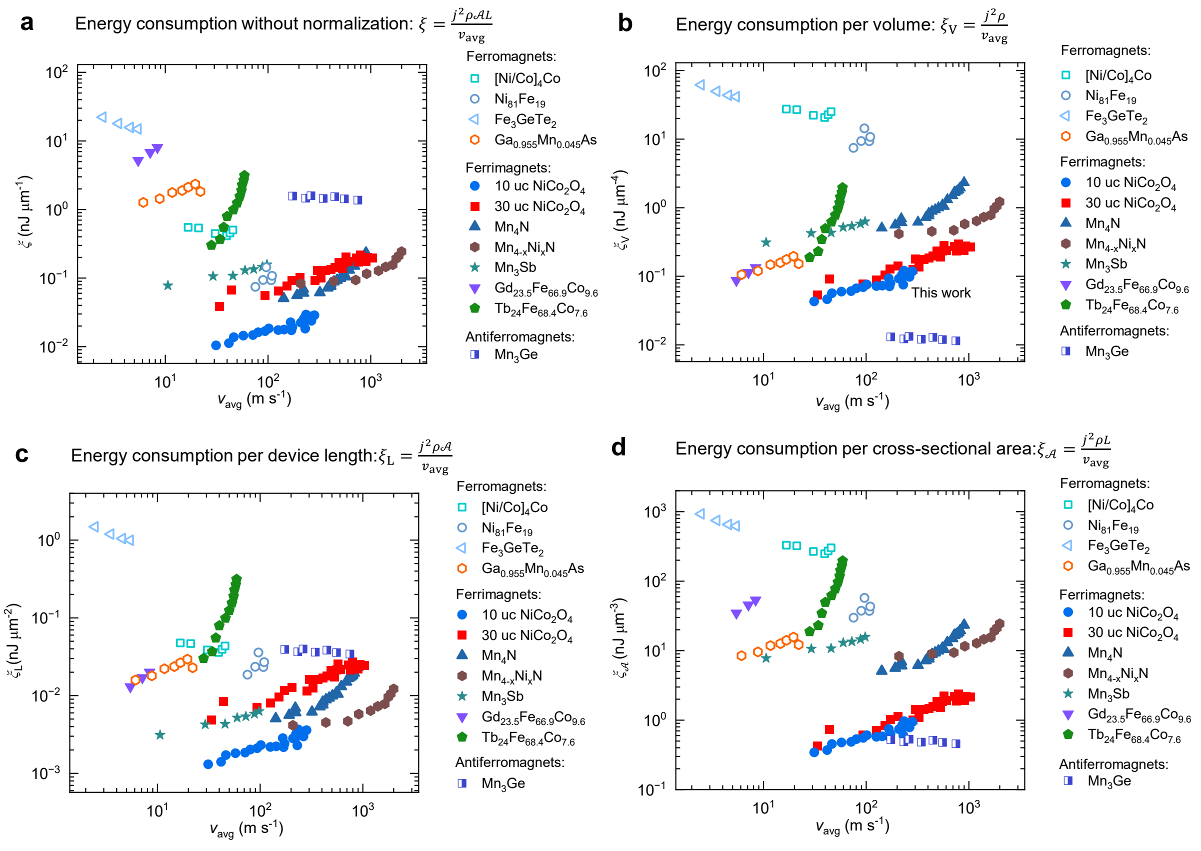

Fig. S8. Energy consumption for 1 μm DW displacement for different normalization factors. **a**, Energy consumption without normalization **b,** Energy consumption per device volume, same as Fig. 5b in the main text. **c**, Energy consumption per device length. **d**, Energy consumption per device cross-sectional area.

Materials	Magnetism	ρ(μΩ ·cm)	References
$[Co/Ni]_4/Co$	FM	~ 50*	[43]
$Ni_{81}Fe_{19}$	FM	~ 50*	[44]
Fe_3GeTe_2	FM	400	[45]
$Ga_{0.955}Mn_{0.045}As$	FM	3000	[46]
$NiCo_2O_4$	FI	810	This work
Mn_4N	FI	180	[3]
$Mn_{4-x}Ni_xN$	FI	~180*	[21]
Mn_3Sb	FI	~100*	[12], [47]
$Gd_{23.5}Fe_{66.9}Co_{9.6}$	FI	~280*	[33], [48]
$Tb_{24}Fe_{68.4}Co_{7.6}$	FI	200*	[49]
Mn_3Ge	AFM	150	[41]

Table S3 Summary of the resistivities used for calculating the ξ_V in Fig. 5 of the main text. Here FM, FI, AFM denote ferromagnet, ferrimagnet, and antiferromagnet, respectively. *For references do not report resistivity values, data from literature sources or reasonable estimations are used for the calculations.

References

1. Tetienne, J. P., Hingant, T., Martínez, L. J., Rohart, S., Thiaville, A., Diez, L. H., Garcia, K., Adam, J. P., Kim, J. V., Roch, J. F., Miron, I. M., Gaudin, G., Vila, L., Ocker, B., Ravelosona, D. & Jacques, V. The nature of domain walls in ultrathin ferromagnets revealed by scanning nanomagnetometry. *Nat. Commun.* **6**, 6733 (2015).
2. Coey, J. M. D. *Magnetism and Magnetic Materials*. (Cambridge University Press, 2009).
3. Gushi, T., Jovičević Klug, M., Peña Garcia, J., Ghosh, S., Attané, J. P., Okuno, H., Fruchart, O., Vogel, J., Suemasu, T., Pizzini, S. & Vila, L. Large Current driven domain wall mobility and gate tuning of coercivity in ferrimagnetic Mn_4N thin films. *Nano. Lett.* **19**, 8716–8723 (2019).
4. Caretta, L., Mann, M., Büttner, F., Ueda, K., Pfau, B., Günther, C. M., Hessing, P., Churikova, A., Klose, C., Schneider, M., Engel, D., Marcus, C., Bono, D., Bagschik, K., Eisebitt, S. & Beach, G. S. D. Fast current-driven domain walls and small skyrmions in a compensated ferrimagnet. *Nat. Nanotechnol.* **13**, 1154–1160 (2018).
5. Siddiqui, S. A., Han, J., Finley, J. T., Ross, C. A. & Liu, L. Current-induced domain wall motion in a compensated ferrimagnet. *Phys. Rev. Lett.* **121**, 57701 (2018).
6. Caretta, L., Oh, S. H., Fakhrul, T., Lee, D. K., Lee, B. H., Kim, S. K., Ross, C. A., Lee, K. J. & Beach, G. S. D. Relativistic kinematics of a magnetic soliton. *Science* **370**, 1438–1442 (2020).
7. Vélez, S., Schaab, J., Wörnle, M. S., Müller, M., Gradauskaite, E., Welter, P., Gutgsell, C., Nistor, C., Degen, C. L., Trassin, M., Fiebig, M. & Gambardella, P. High-speed domain wall racetracks in a magnetic insulator. *Nat. Commun.* **10**, 4750 (2019).
8. Tsukamoto, M., Xu, Z., Higo, T., Kondou, K., Sasaki, K., Asakura, M., Gambardella, P., Miwa, S., Otani, Y., Nakatsuji, S., Degen, C. & Kobayashi, K. Observation of chiral domain walls in an octupole-ordered antiferromagnet. *Phys. Rev. B* **112**, L020404 (2025).
9. Wörnle, M. S., Welter, P., Giraldo, M., Lottermoser, T., Fiebig, M., Gambardella, P., & Degen, C. L. Coexistence of Bloch and Néel walls in a collinear antiferromagnet. *Phys. Rev. B* **103**, 094426 (2021).
10. Jenkins, S., Wagner, T., Gomonay, O., & Everschor-Sitte, K. Revealing ultrafast domain wall motion in Mn_2Au through permalloy capping. *Phys. Rev. B* **109**, 224431 (2024).
11. Ryu, K. S., Thomas, L., Yang, S. H. & Parkin, S. S. P. Chiral spin torque at magnetic domain walls. *Nat. Nanotechnol.* **8**, 527–533 (2013).

12. Filippou, P. C., Jeong, J., Ferrante, Y., Yang, S. H., Topuria, T., Samant, M. G. & Parkin, S. S. P. Chiral domain wall motion in unit-cell thick perpendicularly magnetized Heusler films prepared by chemical templating. *Nat. Commun.* **9**, 4653 (2018).
13. Emori, S., Bauer, U., Ahn, S. M., Martinez, E. & Beach, G. S. D. Current-driven dynamics of chiral ferromagnetic domain walls. *Nat. Mater.* **12**, 611–616 (2013).
14. Avci, C. O., Rosenberg, E., Caretta, L., Büttner, F., Mann, M., Marcus, C., Bono, D., Ross, C. & Beach, G. S. (2019). Interface-driven chiral magnetism and current-driven domain walls in insulating magnetic garnets. *Nat. Nanotechnol*. **14**, 561–566 (2019).
15. Yang, S. H., Ryu, K. S. & Parkin, S. S. P. Domain-wall velocities of up to 750 m s^{-1} driven by exchange-coupling torque in synthetic antiferromagnets. *Nat. Nanotechnol.* **10**, 221–226 (2015).
16. Kato, N., Kawaguchi, M., Lau, Y. C., Kikuchi, T., Nakatani, Y., & Hayashi, M. Current-induced modulation of the interfacial Dzyaloshinskii-Moriya interaction. *Phys. Rev. Lett.* **122**, 257205 (2019).
17. Chureemart, P., Evans, R. F. L., D'Amico, I. & Chantrell, R. W. Influence of uniaxial anisotropy on domain wall motion driven by spin torque. *Phys Rev B* **92**, 054434 (2015).
18. Akosa, C. A., Kim, W., Bisig, A., Kläui, M., Lee, K. & Manchon, A. Role of spin diffusion in current-induced domain wall motion for disordered ferromagnets. *Phys. Rev. B* **91**, 094411 (2015).
19. Xiao, J., Zangwill, A. & Stiles, M. D. Spin-transfer torque for continuously variable magnetization. *Phys. Rev. B* **73**, 054428 (2006).
20. Malozemoff, A. P., & Slonczewski, J. C. Magnetic domain walls in bubble materials: advances in materials and device research. *Academic press.* **IV**, 77-121 (1979).
21. Ghosh, S., Komori, T., Hallal, A., Peña Garcia, J., Gushi, T., Hirose, T., Mitarai, H., Okuno, H., Vogel, J., Chshiev, M., Attané, J. P., Vila, L., Suemasu, T. & Pizzini, S. Current-driven domain wall dynamics in ferrimagnetic nickel-doped Mn_4N films: very large domain wall velocities and reversal of motion direction across the magnetic compensation point. *Nano. Lett.* **21**, 2580–2587 (2021).
22. Chen, X., Zhang, X., Han, M. G., Zhang, L., Zhu, Y., Xu, X. & Hong, X. Magnetotransport anomaly in room-temperature ferrimagnetic $NiCo_2O_4$ thin films. *Adv. Mater.* **31**, 1805260 (2019).

23. Wang, M., Sui, X., Wang, Y., Juan, Y. H., Lyu, Y., Peng, H., Huang, T., Shen, S., Guo, C., Zhang, J., Li, Z., Li, H., Lu, N., N'Diaye, A., Arenholz, E., Zhou, S., He, Q., Chu, Y., Duan, W. & Yu, P. Manipulate the electronic and magnetic states in $NiCo_2O_4$ films through electric-field-induced protonation at elevated temperature. *Adv. Mater.* **31**, 1900458 (2019).
24. Ndione, P. F., Shi, Y., Stevanovic, V., Lany, S., Zakutayev, A., Parilla, P. A., Perkins, J. D., Berry, J. J., Ginley, D. S. & Toney, M. F. Control of the electrical properties in spinel oxides by manipulating the cation disorder. *Adv. Funct. Mater.* **24**, 610–618 (2014).
25. Shen, Y., Kan, D., Lin, I. C., Chu, M. W., Suzuki, I. & Shimakawa, Y. Perpendicular magnetic tunnel junctions based on half-metallic $NiCo_2O_4$. *Appl. Phys. Lett.* **117**, 042408 (2020).
26. Shiino, T., Oh, S., Haney, P., Lee, S., Go, G., Park, B. & Lee, K. Antiferromagnetic domain wall motion driven by spin-orbit torques. *Phys. Rev. Lett.* **117**, 087203 (2016).
27. Gomonay, O., Jungwirth, T. & Sinova, J. High antiferromagnetic domain wall velocity induced by Néel spin-orbit torques. *Phys. Rev. Lett.* **117**, 017202 (2016).
28. Shen, Y., Kan, D., Tan, Z., Wakabayashi, Y. & Shimakawa, Y. Tuning of ferrimagnetism and perpendicular magnetic anisotropy in $NiCo_2O_4$ epitaxial films by the cation distribution. *Phys Rev B* 101, 094412 (2020).
29. Xu, X., Mellinger, C., Cheng, Z. G., Chen, X. & Hong, X. Epitaxial $NiCo_2O_4$ film as an emergent spintronic material: Magnetism and transport properties. *J Appl. Phys.* **132**, 020901 (2022).
30. Thiaville, A., Nakatani, Y., Miltat, J. & Suzuki, Y. Micromagnetic understanding of current-driven domain wall motion in patterned nanowires. *Europhys. Lett.* **69**, 990–996 (2005).
31. Haltz, E., Krishnia, S., Berges, L., Mougin, A. & Sampaio, J. Domain wall dynamics in antiferromagnetically coupled double-lattice systems. *Phys. Rev. B* **103**, 014444 (2021).
32. Kim, K. J., Kim, S. K., Hirata, Y., Oh, S. H., Tono, T., Kim, D. H., Okuno, T., Ham, W. S., Kim, S., Go, G., Tserkovnyak, Y., Tsukamoto, A., Moriyama, T., Lee, K. J. & Ono, T. Fast domain wall motion in the vicinity of the angular momentum compensation temperature of ferrimagnets. *Nat. Mater.* ***16***, 1187-1192 (2017).
33. Okuno, T., Kim, D. H., Oh, S. H., Kim, S. K., Hirata, Y., Nishimura, T., Ham, W. S., Futakawa, Y., Yoshikawa, H., Tsukamoto, A., Tserkovnyak, Y., Shiota, Y., Moriyama, T., Kim, K. J., Lee, K. J. & Ono, T. Spin-transfer torques for domain wall motion in antiferromagnetically coupled ferrimagnets. *Nat. Electron.* **2**, 389–393 (2019).

34. Cai, K., Zhu, Z., Lee, J. M., Mishra, R., Ren, L., Pollard, S. D., He, P., Liang, G., Teo, K. & Yang, H. Ultrafast and energy-efficient spin–orbit torque switching in compensated ferrimagnets. *Nat. Electron.* **3**, 37–42 (2020).
35. Thiaville, A., Nakatani, Y., Piéchon, F., Miltat, J. & Ono, T. Transient domain wall displacement under spin-polarized current pulses. *Eur. Phys. J. B.* **60**, 15–27 (2007).
36. Chauleau, J. Y., Weil, R., Thiaville, A. & Miltat, J. Magnetic domain walls displacement: Automotion versus spin-transfer torque. *Phys. Rev. B.* **82**, 214414 (2010).
37. Thomas, L., Moriya, R., Rettner, C. & Parkin, S. S. P. Dynamics of magnetic domain walls under their own inertia. *Science* **330**, 1810-1813 (2010).
38. Torrejon, J., Martinez, E. & Hayashi, M. Tunable inertia of chiral magnetic domain walls. *Nat. Commun.* **7**, 13533 (2016).
39. Zhang, S. & Li, Z. Roles of nonequilibrium conduction electrons on the magnetization dynamics of ferromagnets. *Phys. Rev. Lett.* **93**, 127204 (2004).
40. Miron, I. M., Moore, T., Szambolics, H., Buda-Prejbeanu, L. D., Auffret, S., Rodmacq, B., Pizzini, S., Vogel, J., Bonfim, M., Schuhl, A. & Gaudin, G. Fast current-induced domain-wall motion controlled by the Rashba effect. *Nat. Mater.* **10**, 419–423 (2011).
41. Wu, M., Chen, T., Nomoto, T., Tserkovnyak, Y., Isshiki, H., Nakatani, Y., Higo, T., Tomita, T.; Kondou, K., Arita, R., Nakatsuji, S. & Otani, Y. Current-driven fast magnetic octupole domain-wall motion in noncollinear antiferromagnets. *Nat. Commun.* **15**, 4305 (2024).
42. Kumar, D., Chung, H. J., Chan, J. P., Jin, T., Lim, S. Ter, Parkin, S. S. P., Sbiaa, R. & Piramanayagam, S. N. Ultralow energy domain wall device for spin-based neuromorphic computing. *ACS. Nano.* **17**, 6261–6274 (2023).
43. Chiba, D., Yamada, G., Koyama, T., Ueda, K., Tanigawa, H., Fukami, S., Suzuki, T., Ohshima, N., Ishiwata, N., Nakatani, Y. & Ono, T. Control of multiple magnetic domain walls by current in a Co/Ni nano-wire. *Appl. Phys. Express.* **3**, 073004 (2010).
44. Hayashi, M., Thomas, L., Rettner, C., Moriya, R., Bazaliy, Y. B. & Parkin, S. S. P. Current driven domain wall velocities exceeding the spin angular momentum transfer rate in permalloy nanowires. *Phys. Rev. Lett.* **98**, 037204 (2007).
45. Zhang, W., Ma, T., Hazra, B. K., Meyerheim, H., Rigvedi, P., Yin, Z., Srivastava, A. K., Wang, Z., Gu, K., Zhou, S., Wang, S., Yang, S. H., Guan, Y. & Parkin, S. S. P. Current-induced domain wall motion in a van der Waals ferromagnet Fe_3GeTe_2. *Nat. Commun.* **15**, 4851 (2024).

46. Yamanouchi, M., Chiba, D., Matsukura, F., Dietl, T. & Ohno, H. Velocity of domain-wall motion induced by electrical current in the ferromagnetic semiconductor (Ga,Mn)As. *Phys. Rev. Lett.* **96**, 096601 (2006).

47. Hayashi, H., Shirako, Y., Xing, Lei., Belik, A. A., Arai, M., Kohno, M., Terashima, T., Kojitani, H., Akaogi, M., & Yamaura, K. Large anomalous Hall effect observed in the cubic-lattice antiferromagnet Mn_3Sb with kagome lattice. *Phys. Rev. B* **108**, 075140 (2023).

48. Okuno, T., Kim K. J., Tono, T., Kim, S., Moriyama, T., Yoshikawa, H., Tsukamoto, A. & Ono, T. Temperature dependence of magnetoresistance in GdFeCo/Pt heterostructure. *Appl. Phys. Express* **9**, 073001 (2016).

49. Ngo, D.-T., Ikeda, K., & Awano, H. Direct observation of domain wall motion induced by low-current density in TbFeCo wires. *Appl. Phys. Express* **4**, 093002 (2011).
